# Tracing evolutionary neurodevelopmental pathways in schizophrenia through the analysis of brain sulcal pits and genomic human accelerated regions

**DOI:** 10.1192/j.eurpsy.2026.10631

**Published:** 2026-07-31

**Authors:** N. Hostalet, M. Berrio, M. Guardiola-Ripoll, L. Cobos-Aumatell, C. Almodóvar-Payá, M. Latorre-Guardia, J. Salavert, E. Inarejos-Clemente, S. Sarró, R. Salvador, K. Im, E. Pomarol-Clotet, M. Fatjó-Vilas

**Affiliations:** 1 FIDMAG Germanes Hospitalàries Research Foundation; 2 Departament de Biologia Evolutiva, Ecologia i Ciències Ambientals, Universitat de Barcelona, Barcelona; 3 Instituto de Salud Carlos III, CIBERSAM, Madrid; 4 Institut d’Investigació Sanitària Pere Virgili; 5 Hospital Universitari Institut Pere Mata, Reus; 6 Freelance computing asssessor; 7 Fundació Hospitalàries Barcelona Nord; 8 Servei de Radiologia, Hospital Sant Joan de Déu, Barcelona, Spain; 9 Fetal-Neonatal Neuroimaging and Developmental Science Center; 10 Division of Newborn Medicine, Department of Pediatrics, Boston Children’s Hospital, Harvard Medical School, Boston, United States

## Abstract

**Introduction:**

Schizophrenia (SZ) is a neurodevelopmental disorder with an evolutionary trade-off for human-specific cognitive abilities. Cortical sulcal patterns, tightly linked to evolution and neurodevelopment, represent potential SZ markers prenatally shaped by sulcal pits, the deepest points of cortical sulci. Human Accelerated Regions (HARs), evolutionary genomic elements, may link evolutionary and developmental factors in SZ, through the influence of their prenatal expression on cortical morphology.

**Objectives:**

i) To test the divergence of SZ sulcal pit patterns relative to healthy controls (HC) across lobes and hemispheres; ii) to evaluate the influence of HARs-related gene expression on this divergence.

**Methods:**

The sample included 426 adults (237 SZ/189 HC) with T1-weighted MRIs (1.5 T). Sulcal pits were extracted from FreeSurfer-derived white matter surfaces. Graphs were created for each lobe and hemisphere and compared between SZ and HC using sulcal pit position (SP-Position), depth (SP-Depth), basin surface area (SP-SAbasin), topology (SP-Topology), and combined features (SP-Combined). Transcriptomic data (BrainSpan) included 179 HARs-related genes across 13, 21, and 37 post-conception weeks. Covariation between transcriptomic data and the differences in sulcal pits patterns was tested by Partial Least Squares Regression models.

**Results:**

SZ patients exhibited reduced pit similarity to HC in: i) the left frontal lobe (SP-Combined) in males, ii) in the right parietal lobe (SP-Topology) in females. HARs gene expression during prenatal stages covariated with group differences in a sex-specific manner.

**Conclusions:**

Sulcal pits emerge as sex-specific early neurodevelopmental markers in SZ. These findings provide the first evidence of HARs-driven transcriptional influence on sulcal pits patterns, supporting the neurodevelopmental and evolutionary component of the disorder.

**Acknowledgements:**

ISCIII: PI20/01002, FI21/00093-NH, CP20/00072-MFV, MV23/00059-NH, co-funded by ERDF/ESF; AGAUR: 2021SGR01475.

**Disclosure of Interest:**

None Declared

